# Performance evaluation of large language models in bladder cancer patient education Q&A: a cross-sectional study

**DOI:** 10.3389/fonc.2026.1909227

**Published:** 2026-09-03

**Authors:** Dian Wan, Youwen Li, Zheng Dong, Chen Dai, Jinghe Ye, Song Li, Sunlu Jiang

**Affiliations:** 1 Department of Urology, Huanggang Central Hospital, Affiliated Huanggang Hospital, Hubei University of Science and Technology, Huanggang, China; 2 College of Clinical Medicine, Hubei University of Science and Technology, Xianning, China; 3 Department of Minimally Invasive Intervention, Hubei Cancer Hospital, Tongji Medical College, Huazhong University of Science and Technology, Wuhan, China

**Keywords:** artificial intelligence, bladder cancer, large language models, patient education, readability

## Abstract

**Background:**

Bladder cancer ranks among the most prevalent urological tumors worldwide, with its global incidence continuing to rise steadily. Although patient education materials (PEMs) play a crucial role in enhancing disease comprehension and supporting joint clinical decision-making, current online resources frequently surpass the readability thresholds recommended for the general public. Large language models (LLMs) hold promise for health communication, yet no systematic assessment has been conducted regarding their feasibility and trustworthiness specifically for bladder cancer patient education.

**Objective:**

This study aimed to systematically benchmark five leading LLMs in producing question-and-answer content for bladder cancer science popularization, with a particular focus on readability, informational quality, and appropriateness for patient education.

**Methods:**

In this cross-sectional simulation study, 20 common patient questions covering five disease domains were compiled. On January 15, 2026, each question was submitted identically to five publicly available LLMs (Doubao, DeepSeek, Kimi, Gemini, and ChatGPT). Readability was evaluated using seven conventional metrics. Two independent pharmacists, blinded to model identity, rated the responses using the Chinese version of the Patient Education Materials Assessment Tool for print materials (C-PEMAT-P) and the Global Quality Score (GQS). Additionally, two independent clinical specialists assessed factual accuracy and alignment with the Chinese Bladder Cancer Diagnosis and Treatment Guidelines (2024 edition) employing a 4-point scale. Cohen’s kappa was used to determine inter-rater reliability.

**Results:**

ChatGPT, DeepSeek, and Doubao outperformed Kimi and Gemini on both C-PEMAT and GQS (all P < 0.001), indicating superior understandability, actionability, and overall quality. Across all models, median C-PEMAT scores ranged from 8 to 10, suggesting broadly acceptable suitability for patient education. Readability varied significantly by content domain, with treatment-oriented texts showing the highest complexity. ChatGPT achieved the best alignment with clinical guidelines. No model produced harmful advice or directly contradicted guideline recommendations. Traditional readability measures correlated weakly with GQS, whereas C-PEMAT showed a moderate positive correlation (r = 0.34).

**Conclusion:**

Current mainstream LLMs demonstrate initial potential for generating educational content on bladder cancer, albeit with considerable heterogeneity across models. Disease-specific evaluation instruments for patient education materials are more effective than general readability formulas in reflecting perceived quality. Our results advocate for a prudent, assistive role of LLMs in health communication under a human-AI collaborative model.

## Introduction

Bladder cancer is one of the most common urological malignancies, with a continuously rising incidence globally (1, 2). In China, 266,887 new cases of genitourinary cancer and 108,589 related deaths were reported in 2021, with bladder cancer accounting for a substantial proportion (3, 4). From 1990 to 2021, the age-standardized incidence rate (ASIR) of genitourinary cancer in China increased significantly by 355.6%, and the ASIR of bladder cancer specifically increased by 9.6% (3, 5). Bladder cancer has multiple subtypes and diverse treatment options, making comprehensive patient education an essential component of clinical care (6).

Health science popularization refers to the process of rendering specialized clinical knowledge into lay-friendly, actionable information that can be readily understood by the general public. For bladder cancer specifically, effective popularization can enhance public awareness of risk factors, facilitate early detection of symptoms — with painless hematuria being the most frequent initial sign — and support patients in comprehending intricate therapeutic choices (1). Studies have shown that well-educated bladder cancer patients demonstrate better involvement in treatment decision-making and higher adherence. However, existing online patient education materials consistently exceed recommended readability levels. A study on online health information for common urologic cancers found that the material was written at a 10th- to 11th-grade level on average, significantly higher than the 6th-grade level recommended by the American Medical Association and above the reading ability of the average US adult (P < 0.01) (7). Another study specifically examining bladder cancer online material found that the American Urological Association (AUA) material had a mean readability of 8.5 (8th–9th grade), whereas the top 50 search engine results had a mean readability as high as 11.7 (11th–12th grade), far above the recommended 6th–7th grade level (8).

Large language models (LLMs) have created novel avenues for distributing medical information. Within healthcare, their applications generally span two domains: (i) public-oriented intelligent assistants for science communication and initial consultation support, and (ii) professional aids for clinicians, assisting with literature searches, drafting of documents, and educational activities. The recent ESMO guidance (ELCAP) acknowledges that LLMs may offer advantages in oncology, including enhanced patient education and symptom control (9, 10).

Large language models (LLMs) offer substantial opportunities for public health education. They can generate accurate, patient-friendly content and serve as scalable tools to democratize access to reliable health information, reduce health literacy disparities, and support patient empowerment. In oncology, the ESMO guidance (ELCAP) highlights that LLMs can improve patient education and symptom management. Recent systematic reviews confirm that with appropriate oversight, LLMs produce readable and sufficiently accurate content for patient education, complementing traditional health communication strategies. Therefore, leveraging LLMs for bladder cancer science popularization aligns with the broader goal of advancing equitable and effective health education (6, 11–13).

Therefore, we formulated the core hypothesis that mainstream LLMs differ significantly in their ability to generate readable, high-quality, and educationally suitable bladder-cancer health content, and that domain-specific assessment tools better reflect user-perceived quality than generic readability formulas. This study aimed to comprehensively assess five mainstream LLMs – Doubao, DeepSeek, Kimi, Gemini, and ChatGPT – in generating bladder-cancer-related Q&A content, evaluating performance across three dimensions: readability, information quality, and educational suitability (14). The findings are intended to inform public health communication, guide clinicians considering AI-assisted patient education, and provide empirical evidence for optimizing LLM deployment in health communication.

## Materials and methods

### Ethical considerations

All data analyzed originated solely from LLM outputs, without any involvement of human or animal participants, nor collection of personal identifiers or biological samples. Under prevailing international norms and relevant Chinese ethical guidelines, such data-centric research without human subjects does not require institutional ethics committee approval.

### Research procedure

On January 15, 2026, two attending urologists (with clinical experience of 3 and 5 years, respectively) derived 20 frequently asked questions from actual patient and caregiver inquiries at a Chinese tertiary hospital (**Table 1**). These questions spanned five thematic domains. An additional patient educator and a bladder cancer patient reviewed the questions to confirm their representativeness. Each of the 20 questions was then entered into five publicly accessible LLMs. All prompts were presented in Simplified Chinese using new chat sessions with cleared memory and disabled web search. Free-tier accounts were employed with default safety configurations. To reduce temporal variability, every question was input once between 9:00 and 11:00 a.m. on the same day. The identical prompt (presented in both English and Chinese) read: “Please answer the following question about bladder cancer in Simplified Chinese at a level suitable for patient education: [question]”.

**Table 1:** Issue list.

Issue list
1. Basic knowledge
1. What is bladder cancer, and what are its main pathological and physiological mechanisms?
2. What are the typical clinical symptoms of bladder cancer?
3. How does bladder cancer spread or metastasize?
4. Does bladder cancer have a genetic component, and which genes are mainly involved?
2. Causes and risk factors
1. What are the main causes of bladder cancer?
2. How do environmental factors affect the development of bladder cancer?
3. Why are smokers more likely to get bladder cancer?
4. Which gene mutations significantly increase the risk of bladder cancer?
3. Diagnosis
1. What are the common methods for diagnosing bladder cancer?
2. How can bladder cancer be diagnosed based on symptoms and lab test results?
3. Are there any specific tumor markers for bladder cancer?
4. How can bladder cancer be distinguished from other diseases that cause blood in the urine?
4. Treatment
1. What are the clinical treatment strategies for bladder cancer?
2. What are the advantages and disadvantages of transurethral resection versus laser enucleation of bladder tumors?
3. What drugs are used for bladder irrigation after surgery, and how do they differ?
4. What are the treatment options after radical cystectomy, and how do neoadjuvant chemotherapy, immunotherapy, and targeted therapy affect prognosis?
5. Prevention and recovery
1. What are the key measures to prevent bladder cancer recurrence after surgery?
2. What are the early rehabilitation strategies for bladder cancer patients after surgery?
3. How can psychological rehabilitation be provided for bladder cancer patients?
4. How should bladder cancer patients be followed up after treatment?

### Accuracy analysis

Factual correctness was evaluated by two independent specialists: a chief physician in medical oncology (>10 years of experience) and an attending physician in the same department (6 years of experience). Using a 4-point concordance scale (0 = contradicts guideline; 1 = omits essential information; 2 = largely consistent; 3 = completely consistent), they rated each LLM response against the Chinese Bladder Cancer Diagnosis and Treatment Guidelines (2024 edition) and the NCCN Bladder Cancer Guidelines (Version 1.2026). The inter-rater agreement was strong (κ = 0.82) (15).

### Readability evaluation

Readability was quantified using multiple established formulas accessed via an online computational tool (https://readabilityformulas.com/), as summarized in **Table 2**. In the absence of a universally accepted gold standard or definitive evidence favoring any single metric, we selected the conventional battery of indices commonly employed in prior research (8, 16–18).

**Table 2:** Readability tools, formulas, and descriptions.

Readability Index	Description	Formula
Gunning FOG (GFOG)	It estimates the number of years of education required for a person to understand a given text.	G=0.4 X (W/S+((C*W) X 100))
Flesch Reading Ease Score(FRES)	It was created to assess the readability of newspapers and is particularly effective for evaluating school textbooks and technical manuals. The scores range from 0 to 100, with higher scores indicating greater ease of reading.	I = (206.835 – (84.6 X (B/W)) – (1.015 X (W/S)))
Flesch–Kincaid grade level (FKGL)	Delineates the academic capacity level imperative for grasping the written material	G = (11.8 X (B/W)) + (0.39 X (W/S)) – 15.59
Simple Measure of Gobbledygook (SMOG)	It measures the number of years of education the average person needs to understand a text.	G=1.0430 X √C + 3.1291
Coleman–Liau (CL) score	Evaluates the educational level required for understanding a text and offers an associated grade level in the US education system.	G = (–27.4004 X (E/100)) + 23.06395
Linsear Write (LW)	Offers an approximate assessment of the academic level needed to comprehend the text.	LW = (R + 3C)/S Result • If >20, divide by 2 •If ≤20, subtract 2, and then divide by 2
Automated readability index (ARI)	Assesses the scholastic rank in American educational institutions needed to be capable of comprehending written material. The greater the number of characters, the more complex the term.	ARI=4.71 X I + 0.5*ASL – 21.43

G=Grade level; B=Number of syllables; W=Number of words; S=Number of sentences; I=Flesch Index Score; SMOG=Simple Measure of Gobbledygook; C=Complex words (≥3 syllables); E=predicted Cloze percentage=141.8401 – (0.214590 X number of characters) + (1.079812*S); C*=Complex words with exceptions including, proper nouns, words made 3 syllables by addition of “ed” or “es”, compound words made of simpler words. ASL=the average number of sentences per 100 words R=the number of words ≤2 syllables.

### Quality assessment

This study utilized the C-PEMAT-P scale (Chinese version of the Patient Education Materials Assessment Tool) and the GQS (Global Quality Score) scale to evaluate the textual answers. The C-PEMAT-P contains 24 items divided into two dimensions: “Understandability” (16 items, including logical organization of information, simplification of professional terminology.) and “Actionability” (8 items, including provision of concrete action guidance, suitability for target populations.) (19). Each item is scored on a 0–1 scale (0 = completely does not meet, 1 = completely meets), with a total score ranging from 0 to 24. A higher score indicates greater accessibility of the material for users. The GQS uses a 1–5 point scale as previously defined.

Two independent urologists rated the responses on January 15, 2026, using the above instruments. Model identity was masked: all 100 outputs (20 questions × 5 models) were randomized and assigned anonymous codes. Disagreements were resolved by a third expert (a chief medical oncologist with 10 years of experience) through consensus discussion, and the final score was the agreed-upon rating. Cohen’s kappa coefficients for both the C-PEMAT-P and GQS scales were greater than 0.75, meeting the standard for excellent agreement.

### Statistical analysis

Normality of continuous variables was examined using the Shapiro-Wilk test. Normally distributed measures (C-PEMAT and GQS scores) are reported as mean ± standard deviation (Mean ± SD) and were compared across groups using one-way analysis of variance (ANOVA). Non-normally distributed variables (readability indices) are presented as median with interquartile range (M, Q1, Q3) and were analyzed using the Kruskal-Wallis H test. Statistical significance was defined as two-sided P < 0.05.

All data analyses and data visualizations were conducted using Python 3.14. The correlation matrix was computed using the Pandas library, and the heatmap was generated using the Seaborn library, where each cell represents the pairwise r value. Color intensity indicates the strength and direction of correlation (red: positive, blue: negative).

## Results

### Normality analysis

C-PEMAT and GQS scores approximated a normal distribution (Shapiro-Wilk P > 0.05), whereas all readability indices (ARI, FRES, GFOG, FKGL, CL, SMOG, LW) were non-normally distributed (P < 0.05).

### Accuracy analysis

Factual accuracy against guidelines (**Table 3**) showed the mean concordance scores (0–3) were: ChatGPT 2.63 ± 0.28, DeepSeek 2.41 ± 0.30, Doubao 2.12 ± 0.49, Kimi 1.75 ± 0.64, Gemini 1.49 ± 0.53. No response contained harmful advice or direct contradictions to guidelines. However, Kimi and Gemini had more omissions of key information.

**Table 3:** Analysis results of different large models.

Variables	Total (n = 100)	Doubao (n = 20)	Deep Seek (n = 20)	Kimi (n = 20)	Gemini (n = 20)	GPT-5.5 (n = 20)	Statistic	P
C-PEMAT score, Mean ± SD	7.75 ± 2.11	7.85 ± 1.69	8.15 ± 1.93	7.05 ± 1.96	6.75 ± 2.55	8.95 ± 1.76	F=3.87	0.006
GQS score, Mean ± SD	2.74 ± 1.35	2.50 ± 1.32	3.30 ± 1.08	2.00 ± 1.08	1.90 ± 0.97	4.00 ± 1.03	F=13.27	<.001
ARI, M (Q1, Q3)	19.00 (16.27, 23.20)	16.57 (13.71, 18.42)	24.91 (20.14, 27.23)	23.20 (21.16, 26.07)	18.43 (17.24, 19.88)	15.00 (12.30, 18.21)	χ²=46.65#	<.001
FRES, M (Q1, Q3)	15.50 (4.00, 30.25)	21.00 (13.75, 33.25)	7.50 (2.00, 15.75)	0.00 (0.00, 9.25)	19.50 (14.00, 29.25)	33.00 (15.50, 48.25)	χ²=31.31#	<.001
GFOG, M (Q1, Q3)	15.70 (13.75, 18.10)	15.30 (13.47, 16.23)	17.45 (14.73, 18.28)	19.40 (17.67, 22.00)	15.35 (13.18, 17.55)	12.75 (9.67, 14.43)	χ²=34.63#	<.001
FKGL, M (Q1, Q3)	17.29 (15.00, 21.20)	15.93 (12.81, 16.91)	21.91 (18.07, 23.31)	21.56 (19.67, 23.44)	17.18 (15.75, 17.95)	13.46 (10.93, 16.37)	χ²=46.98#	<.001
CL, M (Q1, Q3)	16.14 (14.29, 18.38)	15.66 (14.59, 17.77)	15.93 (14.98, 17.59)	19.36 (17.56, 23.42)	16.03 (14.42, 17.05)	14.77 (12.59, 17.58)	χ²=20.51#	<.001
SMOG, M (Q1, Q3)	14.11 (12.43, 16.50)	12.59 (11.48, 14.11)	16.26 (14.11, 18.16)	17.35 (16.05, 18.38)	13.81 (13.52, 15.23)	11.91 (8.92, 13.01)	χ²=48.02#	<.001
LW, M (Q1, Q3)	52.50 (46.75, 60.00)	59.50 (53.75, 62.75)	49.00 (46.00, 56.25)	45.00 (41.75, 49.00)	52.50 (47.00, 54.50)	58.00 (54.75, 62.75)	χ²=31.27#	<.001

### Readability analysis

Inter-model comparisons (**Table 3**) demonstrated significant differences in both C-PEMAT and GQS scores. ChatGPT, DeepSeek, and Doubao achieved significantly higher C-PEMAT scores (8.95 ± 1.76, 8.15 ± 1.93, and 7.85 ± 1.69, respectively) and GQS scores (4.00 ± 1.03, 3.30 ± 1.08, and 2.50 ± 1.32, respectively) than Kimi and Gemini (all P < 0.001). Significant inter-model differences were also observed across multiple readability indices, including ARI, FRES, GFOG, FKGL, SMOG, lexical word ratio (LW), and the Coleman–Liau Index (CL).

Analysis by content topic (**Table 4**) revealed significant differences in C-PEMAT scores across the five topic categories, whereas no statistically significant differences were observed in GQS scores. For readability metrics, materials addressing diagnostic methods, treatment and management, and prevention and prognosis consistently demonstrated higher scores on several reading difficulty indices (such as ARI and FKGL) compared with texts focusing on disease cognition and symptom performance, indicating greater linguistic complexity. The Flesch Reading Ease Score further indicated that disease cognition content exhibited the highest readability, whereas treatment and management content was the most difficult to read. Similar patterns were observed for the Gunning Fog Index, SMOG index, and Coleman–Liau Index.

**Table 4:** Analysis results by content category.

Variables	Total (n = 100)	Disease cognition dimension (n = 20)	Symptom performance dimension (n = 20)	Diagnosis and examination dimension (n = 20)	Treatment and management dimension (n = 20)	Prevention and prognosis dimension (n = 20)	Statistic	P
C-PEMAT score, Mean ± SD	7.75 ± 2.11	6.25 ± 1.52	6.90 ± 1.37	7.45 ± 2.39	8.35 ± 2.08	9.80 ± 1.01	F=12.48	<.001
GQS score, Mean ± SD	2.74 ± 1.35	2.65 ± 1.27	2.95 ± 1.57	3.05 ± 1.43	2.85 ± 1.23	2.20 ± 1.15	F=1.26	0.291
ARI, M (Q1, Q3)	19.00 (16.27, 23.20)	18.31 (12.36, 21.11)	18.20 (16.81, 22.76)	19.27 (16.97, 24.71)	19.26 (16.50, 21.41)	19.28 (17.05, 24.42)	χ²=2.76#	0.600
FRES, M (Q1, Q3)	15.50 (4.00, 30.25)	28.00 (12.50, 50.25)	20.00 (10.00, 36.75)	8.50 (1.50, 28.50)	12.00 (0.00, 19.25)	11.50 (6.25, 28.00)	χ²=12.77#	0.012
GFOG, M (Q1, Q3)	15.70 (13.75, 18.10)	14.10 (11.38, 15.88)	14.25 (12.23, 15.60)	17.20 (15.00, 18.93)	17.50 (14.80, 19.20)	16.95 (14.95, 18.98)	χ²=17.54#	0.002
FKGL, M (Q1, Q3)	17.29 (15.00, 21.20)	16.81 (10.66, 18.98)	16.52 (14.57, 21.20)	17.49 (16.15, 23.20)	17.29 (16.25, 21.25)	17.93 (15.12, 21.88)	χ²=5.63#	0.229
CL, M (Q1, Q3)	16.14 (14.29, 18.38)	14.28 (12.91, 16.73)	15.86 (14.04, 17.14)	15.94 (14.16, 18.41)	17.56 (16.46, 20.95)	17.48 (15.60, 18.47)	χ²=15.98#	0.003
SMOG, M (Q1, Q3)	14.11 (12.43, 16.50)	13.52 (9.86, 15.27)	13.52 (11.39, 15.49)	14.67 (12.91, 17.24)	14.45 (12.91, 17.00)	15.23 (12.61, 17.35)	χ²=7.87#	0.096
LW, M (Q1, Q3)	52.50 (46.75, 60.00)	56.00 (47.00, 63.00)	51.50 (46.75, 55.25)	54.00 (45.50, 61.00)	51.00 (43.00, 57.00)	54.00 (47.75, 58.50)	χ²=3.43#	0.489

### Quality analysis

**Figure 1:**
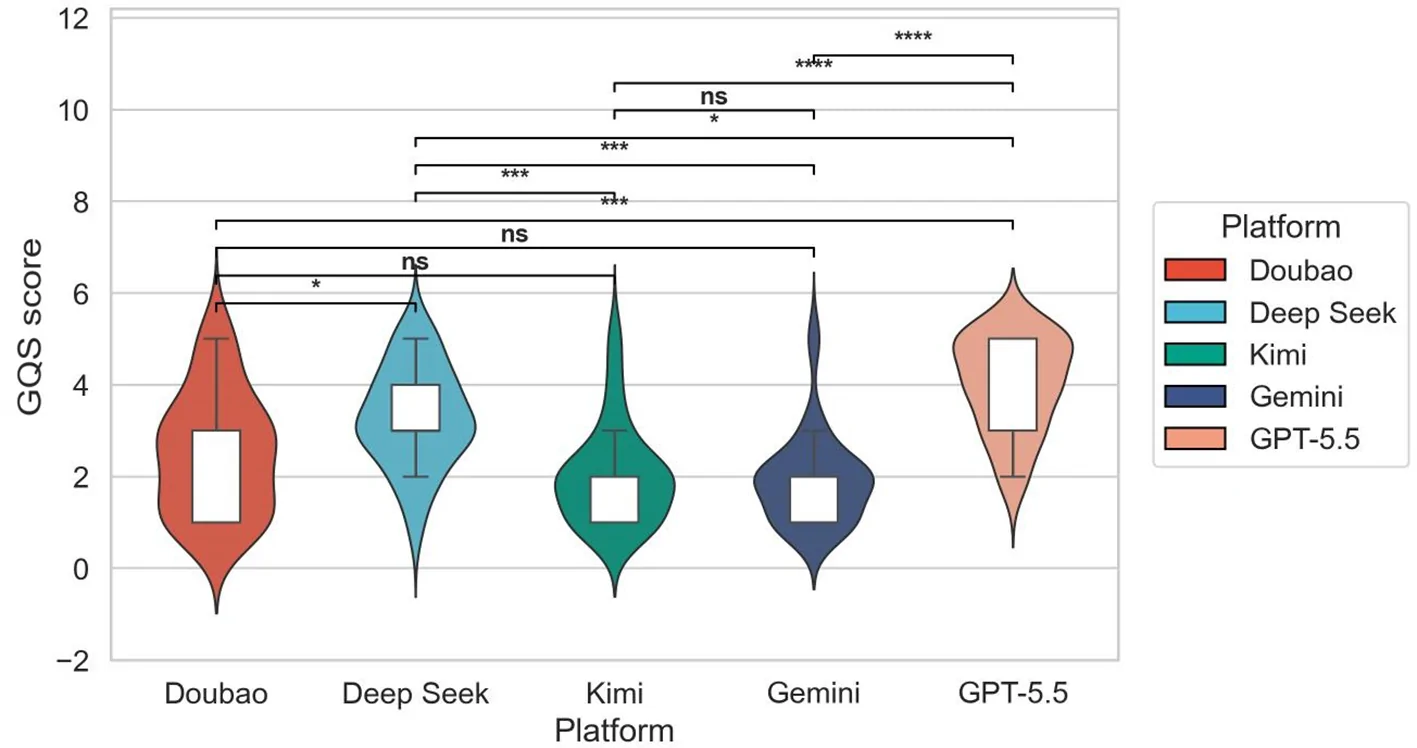
GQS scores of different large models.

Significant differences in GQS scores were observed among the evaluated models (**Figure 1**). ChatGPT exhibited the highest median GQS score with the compact distribution, indicating a strong ability to consistently generate high-quality health information. DeepSeek demonstrated a slightly lower but still relatively high median score with a compact distribution, suggesting competitive performance. Kimi and Gemini showed similar median GQS scores; however, Gemini displayed a wider score distribution with the presence of low-score outliers, indicating variability in output quality. Doubao showed the lowest relative median GQS score and a more dispersed distribution compared to ChatGPT and DeepSeek.

**Figure 2:**
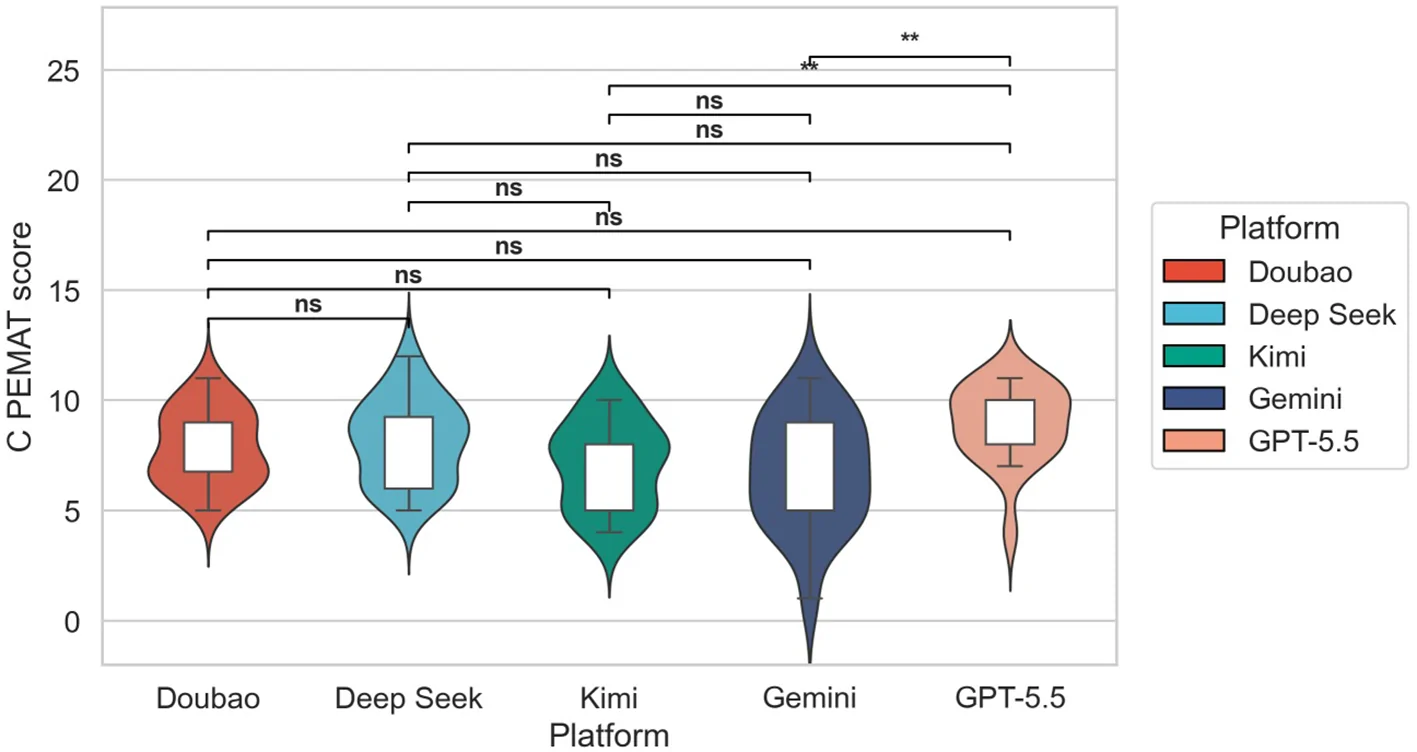
C-PEMAT scores of different large models.

With respect to C-PEMAT scores (**Figure 2**), the median values for all models fell within the range of 7–9 points, suggesting that mainstream LLMs generally possess a baseline capability to generate structurally clear and logically coherent patient education materials. Among the models, ChatGPT achieved the highest median score with the most concentrated distribution, indicating the most robust overall performance. DeepSeek and Kimi followed, whereas Doubao demonstrated the lowest median C-PEMAT score among ChatGPT, DeepSeek, and Doubao, yet still outperformed Kimi and Gemini. Its bimodal distribution reflected instability in understandability and actionability.

### Correlation analysis

**Figure 3:**
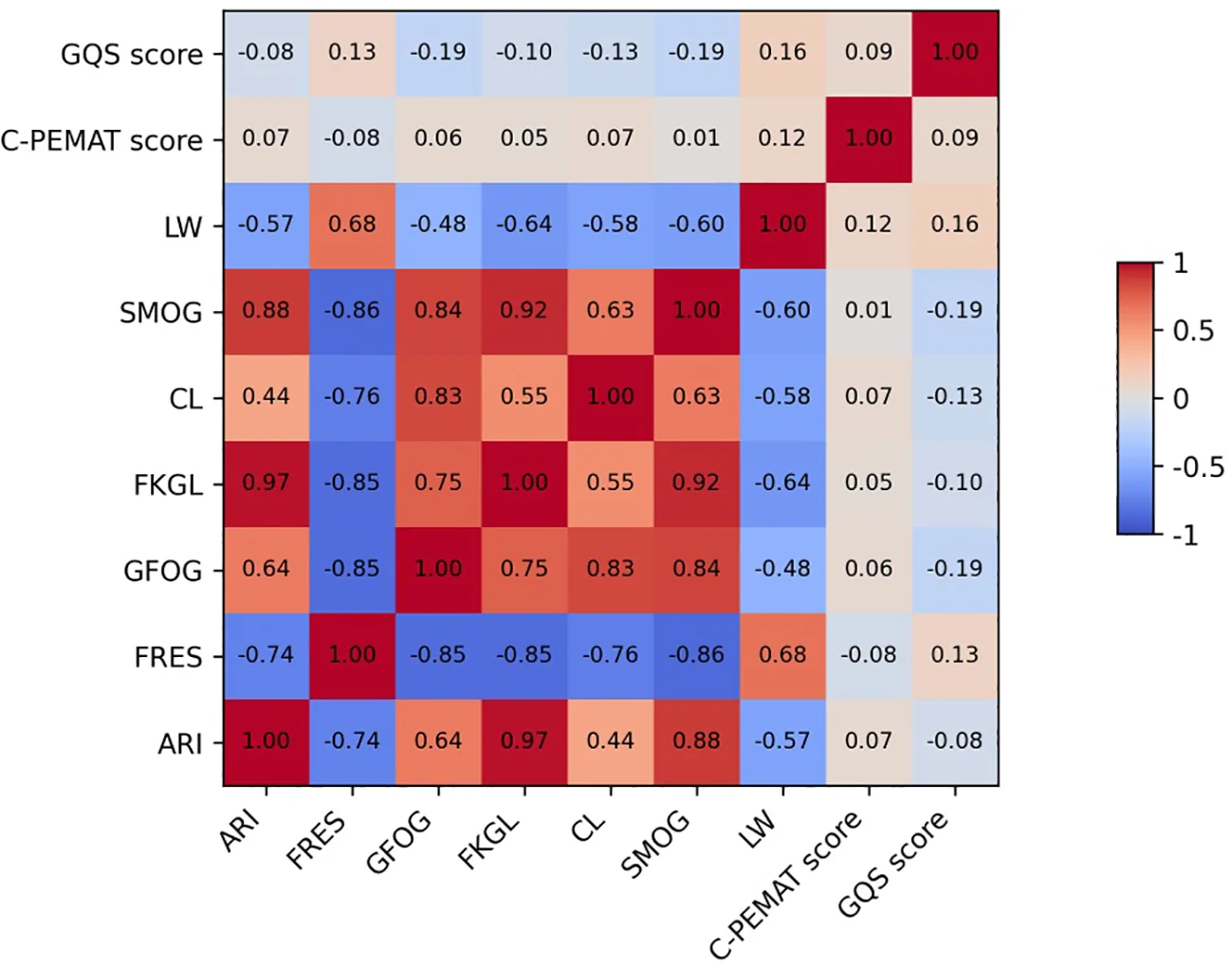
Heatmap of correlations among different indicators.

Correlation analyses were conducted to explore relationships among readability indices, C-PEMAT scores, and user-perceived quality as measured by GQS (**Figure 3**). Readability ease indicators, such as the Flesch Reading Ease Score, showed a weak positive correlation with GQS (r = 0.09) and strong negative correlations with several reading difficulty indices (GFOG, r = –0.86; SMOG, r = –0.79). Traditional reading difficulty formulas (ARI, FKGL, SMOG, and related indices) were highly intercorrelated (r > 0.89) but demonstrated weak or negligible correlations with GQS (e.g., ARI vs. GQS, r = 0.03). In contrast, the C-PEMAT score exhibited a moderate positive correlation with GQS (r = 0.34). Lexical richness, as measured by LW, was positively correlated with FRES (r = 0.68) and negatively correlated with multiple reading difficulty indices.

## Discussion

### Principal findings and scientific contribution

This study presents a systematic, multidimensional evaluation of five mainstream large language models (LLMs) for bladder-cancer-related health communication. Three principal observations warrant emphasis. First, model identity was strongly associated with patient-education suitability and overall informational quality, with ChatGPT, DeepSeek, and Doubao generally outperforming Kimi and Gemini on both C-PEMAT and GQS. Second, content category influenced readability more markedly than it influenced C-PEMAT or GQS scores: materials concerning treatment/management and diagnosis/examination exhibited greater linguistic complexity, whereas those covering basic disease knowledge and symptomatology were consistently easier to read. Third, conventional readability indices correlated only weakly with C-PEMAT and GQS. Collectively, these findings indicate that model selection and content domain affect different dimensions of LLM-generated health information, and that linguistic simplicity, educational suitability, and overall quality should not be regarded as interchangeable. The principal novelty of this work lies in its concurrent assessment of model type, content category, output distribution, educational suitability, and formula-based readability within a bladder-cancer-specific communication framework.

### Inter-model differences in relation to previous evidence

The substantial inter-model differences observed in this study are consistent with a growing body of evidence indicating that the quality of LLM-generated medical information varies across platforms and evaluation instruments (20–22). Our observation that ChatGPT achieved the highest C-PEMAT and GQS scores aligns with the findings of Sivaramakrishnan et al., who reported that ChatGPT-4.0 exhibited superior understandability in generating dental patient education materials, although actionability remained suboptimal across models (23). These converging results suggest that certain architectures may be more adept at information organization, patient-oriented explanation, and provision of explicit behavioral guidance. Nonetheless, the relative performance of individual models has varied considerably across clinical domains, arguing against the notion of a universally superior model.

This task dependence becomes particularly apparent when comparing our results with the study form Balaban, who evaluated Gemini 2.5 Flash, ChatGPT-5.1, and DeepSeek-R1 on the Turkish Pharmacy Specialty Examination (24). Gemini attained the highest numerical accuracy, followed by ChatGPT and DeepSeek, though differences in overall accuracy and readability did not reach statistical significance. In our evaluation, by contrast, Gemini performed less favorably, and significant inter-model differences were detected by C-PEMAT and GQS. These findings are not necessarily incompatible. The pharmacy examination study primarily assessed the retrieval and application of professional knowledge under standardized testing conditions, whereas our investigation focused on whether free-text responses were understandable, actionable, and suitable for patient education. Variation in language, model version, clinical domain, prompt design, response format, and evaluation instruments may further contribute to the discrepant rankings. This comparison underscores the importance of context-specific LLM evaluation and cautions against extrapolating examination-based performance to patient-facing communication.

Our results also complement prior evidence from urological oncology (10). Shah reported that ChatGPT-generated materials surpassed conventional educational resources in overall quality but demanded a substantially higher reading level. When ChatGPT was explicitly instructed to simplify its responses, readability improved while quality was largely preserved. This quality–readability trade-off resonates with our finding that strong educational quality does not automatically confer linguistic accessibility. Collectively, these studies suggest that LLM performance is inherently multidimensional: a model may generate comprehensive, well-structured content while simultaneously producing language that exceeds the comprehension capacity of certain patient populations.

The observed performance disparities may plausibly arise from differences in training corpora, instruction tuning, alignment methodologies, response-planning capabilities, and adaptation to patient-oriented language. However, because the architectures and training procedures of several evaluated models remain proprietary, our study cannot definitively attribute the observed differences to specific technical features. These interpretations should therefore be regarded as hypotheses meriting further investigation rather than as established causal relationships.

Output consistency is another consideration for clinical deployment. Within the present dataset, ChatGPT and DeepSeek exhibited narrower interquartile ranges and fewer low-scoring outliers on C-PEMAT and GQS, suggesting more stable performance across the question set (25, 26). In contrast, the broader distributions and occasional low-quality outputs observed for other models indicate that satisfactory mean performance does not preclude the generation of unsuitable individual responses. Nonetheless, distributional patterns observed in a single experiment should not be equated with temporal reproducibility, as LLM outputs may vary with repeated queries and subsequent model updates. Longitudinal, repeated-generation studies are needed before conclusions regarding long-term reliability can be drawn.

### Content-dependent readability and its implications

Beyond model-related variation, readability differed substantially across content categories. Treatment/management and diagnosis/examination generated more linguistically demanding responses than disease knowledge and symptom-related topics. This pattern is clinically plausible, as therapeutic and diagnostic explanations frequently necessitate specialized terminology, stepwise procedural descriptions, risk–benefit comparisons, and conditional recommendations (27). Comparable topic-dependent effects have been documented in urological oncology, where treatment-related materials remained more difficult to read than general information even after prompting for simplification. The present findings thus extend prior evidence by demonstrating that the intrinsic communicative demands of the subject matter may shape readability across multiple LLMs (10).

Notably, content category was not significantly associated with C-PEMAT or GQS. This dissociation carries important implications. It indicates that linguistically complex material may nevertheless be logically structured, adequately comprehensive, and potentially actionable. Conversely, a response composed of short sentences and familiar vocabulary may remain educationally inadequate if it omits essential information or fails to provide clear guidance. The absence of content-category differences in C-PEMAT and GQS should not, however, be interpreted as evidence of uniform accuracy or clinical safety across topics, as these instruments do not independently assess factual correctness.

These observations support content-adaptive communication strategies. For diagnosis- and treatment-related topics, LLM prompts should encourage segmentation into discrete information units, explicit definition of technical terms, stepwise explanations, and clear differentiation of benefits, risks, and indications for professional consultation. Analogies may enhance accessibility but require clinical review to ensure that simplification does not introduce misleading interpretations. For symptom recognition, prevention, and daily care guidance, concise language and direct action-oriented statements may be more appropriate (11, 28).

### Divergence between readability and educational quality

One of the more clinically salient findings of this study was the weak association between conventional readability indices and C-PEMAT or GQS. Traditional readability formulas primarily estimate surface-level linguistic complexity based on sentence length, word length, and syllable count. They do not directly assess logical organization, factual completeness, actionability, communication of uncertainty, or alignment with patients’ informational needs (29). Consequently, improving a formula-based readability score does not necessarily translate into more effective patient education.

In the present study, C-PEMAT and GQS detected significant inter-model differences that were less consistently captured by conventional readability indices. This does not establish the universal superiority of one instrument type over another, as they measure fundamentally different constructs. Rather, it suggests that patient-education-oriented instruments may offer greater discriminatory value when the goal is to assess whether materials are understandable, actionable, and practically useful. Conventional readability indices remain useful for flagging excessive linguistic complexity, but they should be interpreted alongside educational-quality instruments, clinical accuracy assessments, and—where feasible—direct patient testing.

The positive association between GQS and response length further illustrates the tension between completeness and accessibility. Longer responses may receive higher quality ratings because they incorporate essential qualifications, contraindications, and stepwise recommendations. However, this association should not be construed as evidence that increasing response length intrinsically improves quality. Excessive detail may impose cognitive burden, whereas excessive brevity may omit clinically important information. Similarly, technical terminology cannot always be eliminated without sacrificing precision. The more appropriate strategy is to introduce necessary terms selectively and explain them in plain language (12).

### Clinical and public health implications

The results support the cautious integration of LLMs as drafting tools for bladder cancer education rather than as autonomous sources of patient guidance. ChatGPT, DeepSeek, and Doubao may be suitable candidates for generating initial drafts under the conditions evaluated in this study. However, their relative performance reflects a time- and task-specific comparison rather than a general endorsement. All generated materials should undergo professional review, with particular attention to diagnosis, treatment selection, adverse effects, contraindications, uncertainty, and indications for urgent medical consultation.

A practical implementation pathway might encompass LLM-assisted drafting, verification against current clinical guidelines, assessment using patient-education-oriented instruments, adaptation to the target population’s health-literacy level, and final review by clinicians or health educators. For populations with limited health literacy, prompting strategies should specify the intended reading level, request explanations of unavoidable medical terminology, and prioritize actionable information. LLMs should thus augment, rather than replace, clinician-led education and shared decision-making.

## Limitations

This study has several limitations that warrant consideration. First, the use of 20 questions across five domains and five LLMs limits the generalizability of our findings to other models or real-world patient scenarios. Second, our reliance on expert-based evaluations, rather than patient-centered outcomes, and the absence of real-time clinician feedback, constrain the direct clinical applicability of our results. Third, individual patient heterogeneity—spanning health literacy, age, and cultural background—was not addressed, limiting the development of personalized content adaptation strategies and the generalizability across diverse populations. Fourth, the single-day data collection window does not capture intra-model variability or post-update performance shifts. Although we standardized query timing, session initialization, and disabled web search to minimize such effects, the generalizability of these cross-sectional findings to other time points or updated model versions remains constrained. Fifth, conventional readability formulas were developed for English texts and may not directly translate to Chinese medical content. While these indices have been used as comparative references in recent Chinese-language LLM studies, their absolute values should be interpreted with caution. Sixth, the sample size of 20 questions, while consistent with methodological precedents in this field, was selected without formal power calculation, formally powered studies are warranted. Finally, although factual accuracy was benchmarked against Chinese and international guidelines, the findings may not generalize to other national or regional treatment frameworks.

## Future directions

Based on our findings, we propose three priority areas for future investigation. First, subsequent studies should broaden the range of LLMs evaluated, including newly emerging models and medical-domain fine-tuned variants, while also systematically refining prompt engineering and few-shot learning approaches to improve output quality on complex medical topics (26).

Second, prospective validation in real-world clinical and community environments is needed to determine how LLM-generated educational materials affect patient understanding, health behaviors, and clinical endpoints (30).

Third, future efforts should focus on constructing robust implementation frameworks that incorporate automated fact-checking against evidence-based guidelines, culturally adapted assessment instruments, and well-defined boundaries for human-AI collaboration (27, 31).

## Conclusion

In summary, our findings demonstrate that proper model selection is crucial for generating high-quality, educationally appropriate AI-based health content, while content topic strongly shapes readability. By elucidating the interplay between model attributes, topic difficulty, and communication effectiveness, this study offers actionable guidance for the responsible use of LLMs in patient education and public health communication. Future developments should balance informational completeness with adaptive simplification, aiming to deliver accurate, accessible, and personalized AI-assisted health education tailored to individual needs.

## Data Availability

The original contributions presented in the study are included in the article/supplementary material, further inquiries can be directed to the corresponding author/s.
